# Water availability not fruitfall modulates the dry season distribution of frugivorous terrestrial vertebrates in a lowland Amazon forest

**DOI:** 10.1371/journal.pone.0174049

**Published:** 2017-03-16

**Authors:** Omar Stalin Landázuri Paredes, Darren Norris, Tadeu Gomes de Oliveira, Fernanda Michalski

**Affiliations:** 1 Laboratório de Ecologia e Conservação de Vertebrados, Universidade Federal do Amapá, Macapá, Amapá, Brazil; 2 Programa de Pós-Graduação em Biodiversidade Tropical, Universidade Federal do Amapá, Macapá, Amapá, Brazil; 3 Departamento de Biologia, Centro de Ciências Exatas e Naturais, Universidade Estadual do Maranhão, São Luís, Maranhão, Brazil; 4 Instituto Pró-Carnívoros, Atibaia, São Paulo, Brazil; Università degli Studi di Napoli Federico II, ITALY

## Abstract

Terrestrial vertebrate frugivores constitute one of the major guilds in tropical forests. Previous studies show that the meso-scale distribution of this group is only weakly explained by variables such as altitude and tree basal area in lowland Amazon forests. For the first time we test whether seasonally limiting resources (water and fallen fruit) affect the dry season distribution in 25 species of terrestrial vertebrates. To examine the effects of the spatial availability of fruit and water on terrestrial vertebrates we used a standardized, regularly spaced arrangement of camera-traps within 25km^2^ of lowland Amazon forest. Generalized linear models (GLMs) were then used to examine the influence of four variables (altitude, distance to large rivers, distance to nearest water, and presence vs absence of fruits) on the number of photos on five functional groups (all frugivores, small, medium, large and very large frugivores) and on seven of the most abundant frugivore species (*Cuniculus paca*, *Dasyprocta leporina*, *Mazama americana*, *Mazama nemorivaga*, *Myoprocta acouchy*, *Pecari tajacu* and *Psophia crepitans*). A total of 279 independent photos of 25 species were obtained from 900 camera-trap days. For most species and three functional groups, the variation in the number of photos per camera was significantly but weakly explained by the GLMs (deviance explained ranging from 6.2 to 48.8%). Generally, we found that the presence of water availability was more important than the presence of fallen fruit for the groups and species studied. Medium frugivores, large-bodied frugivores, and two of the more abundant species (*C*. *paca* and *P*. *crepitans*) were recorded more frequently closer to water bodies; while none of the functional groups nor the most abundant species showed any significant relationship with the presence of fallen fruit. Two functional groups and two of the seven most common frugivore species assessed in the GLMs showed significant results with species-specific responses to altitude. Our findings provide a more detailed understanding of how frugivorous vertebrates cope with periods of water and fruit scarcity in lowland Amazon forests.

## Introduction

Environmental features such as water availability and food resources may influence the spatial distribution of wildlife to varying degrees depending on species-specific factors. For example, in subtropical semi-arid regions many species of mammals showed a positive association between occupancy patterns and permanent water sources [1–5]. Similarly, a preference for areas close to water bodies has also been documented in tropical forests for some species of terrestrial birds [6, 7].

In tropical forests, frugivorous species represent the majority of the mammalian and avian biomass [8] and depend on both unripe and ripe fruits as well as some species of flowers as key diet resources [9], with the degree of dependency related with the specificity of their diet [6, 10–12]. However, the availability of resources (e.g. food and water) is not constant in tropical forests and has been found to be extremely variable in both space and time [13–15]. Even continuous tracts of tropical forests can show distinct vertebrate species distributions [16], a phenomena that could be related with habitat suitability and resource availability. Although fruit production varies in space and time, a period of fruit scarcity exists everywhere [17]. Some studies use precipitation as indicator of food availability for frugivores, with the dry season a period of limited food and water availability for Amazon vertebrate frugivores [14, 15, 18, 19].

Seasonal shortages are sufficient to cause several effects in a local frugivore community [14]. A species’ response to fruit and water availability may be influenced by its life-history traits (e.g., dietary guild, body mass, home range size) and competitive pressure [2–4, 20, 21]. Species traits are also used to explain variations on species’ abundances in responses to environmental variables and anthropogenic disturbances [5, 21–27].

Studies on the relationships between life-history traits and water and fruit dependency are still scarce for neotropical terrestrial vertebrates. Although numerous studies have monitored vertebrates in lowland Amazonia [7, 24, 25, 28], the relative importance of different resources (e.g. water and fruit) have not been quantified for terrestrial vertebrates. As Amazonia holds both, the highest diversity of terrestrial and aquatic frugivorous vertebrates [11, 19] and the widest spectrum of morphological fruit types anywhere on Earth [29] it is surprising that more studies on this topic are not found.

The differences in temperature and precipitation regimes predicted by climate change models in the coming decades and their possible influence on resource availability (e.g., fruits and water) [1] may influence population parameters of frugivore species. There is a need to understand the ecological factors affecting the distribution of different species and obtain basic information of the species-specific responses to cope with water and fruit scarcity. This information may inform models for understanding or predicting the potential impact of anthropogenic and climate change on the species in the future, including the potential resilience of frugivores to disturbances [2].

In this study we used camera-traps to survey mid-sized and large- bodied vertebrates with a standardized sampling regime that has been utilized in other tropical studies to survey terrestrial vertebrates within a 25 km^2^ area [7]. Here we focused on evaluating the spatial effects of (1) fruit availability, (2) water availability and (3) altitude on vertebrate frugivores during the peak of the dry season, a period of reduced resources in eastern Amazonia. We included altitude as a proxy for biological variables as it affects soil, water availability, climate and other abiotic and biotic variables [30–34] and has also been shown to be important for the vertebrate groups in the study area [7]. Additionally, we examined the importance of species life-history traits in the responses to these environmental variables. We predict that both the presence of fallen fruit and water availability would affect functional groups and species of terrestrial frugivores. To test these predictions we evaluated whether water and fruit availability act with altitude to explain abundance patterns in terrestrial vertebrates during the dry season in a continuous forest site.

## Materials and methods

### Ethics statement

Data collection used non-invasive, remotely activated camera traps and did not involve direct contact or interaction with animals, thus no ethical approval was required. Fieldwork was conducted under research permit number IBAMA/SISBIO 47859–1 to DN and FM, issued by the Instituto Chico Mendes de Conservação da Biodiversidade (ICMBio).

### Study area

**Fig 1:**
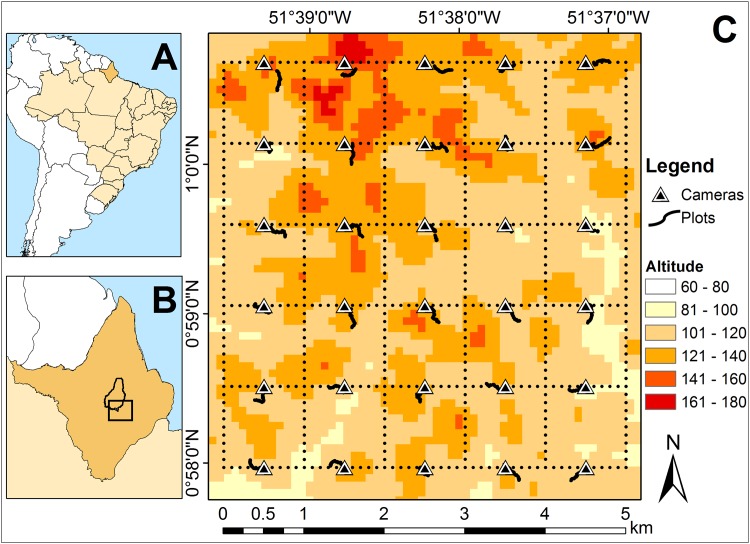
Location of the study region in the Amapá National Forest (ANF), Amapá State, eastern Brazilian Amazon. (A) Amapá State in Brazil; (B) ANF (polygon) in Amapá State; (C) Altitude (m) across the grid system (dotted lines), non-linear plots placed along topographic contours and linear plots along the trails (solid back lines) where the study was conducted. Camera traps were placed at 30 regularly spaced sample points (triangles).

This study was conducted in Amapá National Forest (Floresta Nacional Amapá –hereafter ANF), a sustainable-use protected area of approximately 412,000 ha, centered in the state of Amapá in north-eastern Brazilian Amazonia (0°55’29”N, 51°35’45”W, Fig 1) [35]. The ANF consists of continuous tropical rainforest vegetation, predominantly never-flooded closed canopy ‘‘terra firme” forest [35].

ANF currently experiences low levels of anthropogenic perturbation (there has been no mechanized logging within the boundaries of ANF) and is part of a large (> 4 million hectares) connected group of protected areas [35] that maintain both continuous undisturbed forests and the complete regional community of medium-sized and large-bodied vertebrates [7].

The regional climate is classified by Köppen-Geiger as Am (Equatorial monsoon) [36], with an annual rainfall greater than 2000 mm [37]. The driest months are September to November (total monthly rainfall < 150 mm) and the wettest months from February to April (total monthly rainfall > 300 mm) [37] (S1 Fig).

### Sampling design

Data were collected between October and December 2015, months that historically represent the peak of the dry season in the study area (monthly mean ± SD = 81 mm ± 74 mm, range = 13 mm– 268 mm, for 2013 to 2015) [37] (S1 Fig). Our study months were particularly dry compared with previous years, with a total rainfall of 124 mm (monthly range = 13 mm– 89 mm) between October-December [37] (S1 Fig). Our research was conducted within a 25 km^2^ RAPELD grid (RAP surveys in the Long-term Ecological Research Sites whose Brazilian acronym is PELD, hence RAPELD) of the Brazilian Program for Biodiversity Research (PPBio) [38, 39] (Fig 1). The RAPELD grid is a standardized arrangement of trails and permanent plots [39]. The current study used the 30 permanent regularly spaced sample points and plots that are distributed at 1 km intervals along the east-west trails in the RAPELD grid [7, 38, 39] (Fig 1). This sample size and arrangement has been shown to be generally robust and representative for quantifying meso-scale spatial patterns in lowland Amazon biodiversity [40].

### Vertebrate sampling

We used camera traps equipped with infrared triggers (Bushnell Trophy Cam, 8MP, Overland Park, KS, USA) to sample terrestrial vertebrates in the RAPELD grid. Due to financial constraints we did not have sufficient cameras to survey all 30 points simultaneously. Thus, cameras were placed at 20 points for 30 consecutive days then immediately transferred to the remaining 10 points. This division of study areas into blocks, with cameras not operating simultaneously within an area due to logistical constrains, is a common practice in studies with camera traps [7, 41–44]. As the sampling was conducted over a relatively short period of time, we assume that this division does not introduce any systematic bias. All cameras were unbaited, installed at 30–40 cm above the ground and facing the trails to ensure the capture of a wide spectrum of vertebrates (from small to large-bodied species). Cameras were deployed for 30 days, functioning continuously (24 hours a day). We configured cameras to film for 40 seconds post-activation, with intervals of 15 seconds between videos, with date-time stamp enabled.

In order to estimate the relative abundance of vertebrates, we considered as independent, videos with over a 30 min interval, when the same species was recorded during the same day on the same camera [7, 45, 46]. This minimum 30-min interval reduces the temporal dependence between camera trap detections [45] and has been widely used in studies with camera traps [47–49]. Vertebrates were identified using field guides of mammals and birds [50–52]. All identifications were double-checked by two researchers with more than 10 years experience (FM and DN).

### Species traits

We conducted a literature survey to obtain two morphological and ecological traits (trophic guild and body mass) for the 25 vertebrates studied (Table 1). All 25 vertebrates were classified into frugivores and non-frugivores based on previously published data (Table 1). We grouped mammals and birds into small species (< 1 kg), medium species (1–5 kg), large species (5–15 kg) and very large species (>15 kg) according to the average body size of adult individuals [26, 53] (Table 1). Finally, we defined five functional groups as follows (Table 1): (i) All frugivores (all frugivores from all body sizes), (ii) Small frugivores (frugivores < 1 kg), (iii) Medium frugivores (frugivores with 1–5 kg), (iv) Large frugivores (frugivores with 5–15 kg) and (v) Very large frugivores (frugivores > 15 kg).

**Table 1:** Trophic guild, body mass, functional group, number of occupied sites, relative abundances and number of independent videos (Detections) for all 25 species examined.

Class/Order/Family	Species	Trophic Guild*	Body Mass (kg)	Functional group	Occupied Sites	RA**(Detections)
**Birds**						
Gruiformes						
Psophiidae	*Psophia crepitans*	Fr/In [54, 55]	1.50 [24]	Medium frugivore	21	0.61 (55)
Cracidae	*Crax alector*	Fr/Sp [24]	3.40 [24]	Medium frugivore	4	0.07 (6)
Tinamiformes						
Tinamidae	*Crypturellus erythropus*	In/Fr [54]	0.42 [54]	Small frugivore	4	0.07 (6)
Tinamidae	*Tinamus major*	Sp/Fr/In [54]	1.20 [24]	Medium frugivore	4	0.04 (4)
**Mammals**						
Didelphimorphia						
	*Didelphis marsupialis*	In/Fr/Vp [28]	1.05 [28]	Medium frugivore	3	0.03 (3)
Artiodactyla						
Cervidae	*Mazama americana*	Fr/Fo [28]	30.0 [28]	Very large frugivore	9	0.36 (32)
	*Mazama nemorivaga*	Fr/Fo [28]	18.0 [28]	Very large frugivore	9	0.18 (16)
Tayassuidae	*Pecari tajacu*	Sp/Fr/Vp [28]	25.0 [28]	Very large frugivore	14	0.49 (44)
Perissodactyla						
Tapiridae	*Tapirus terrestris*	Fr/Fo [28]	150.0 [28]	Very large frugivore	4	0.07 (6)
Carnivora						
Canidae	*Speothos venaticus*	Vp [28]	6.32 [56]	-	1	0.01 (1)
Felidae	*Leopardus pardalis*	Vp [28]	11.90 [24]	-	2	0.09 (8)
	*Leopardus wiedii*	Vp [28]	3.25 [24]	-	1	0.01 (1)
	*Puma concolor*	Vp [28]	51.60 [24]	-	2	0.02 (2)
	*Panthera onca*	Vp [28]	80.00 [24]	-	1	0.01 (1)
Mustelidae	*Eira barbara*	Fr/In/Vp [28]	4.80 [28]	Medium frugivore	2	0.02 (2)
Procyonidae	*Nasua nasua*	In/Vp/Fr [28, 57]	3.10 [28]	Medium frugivore	1	0.01 (1)
	*Procyon cancrivorus*	In/Vp/Fp/Fr [28, 57]	6.93 [56]	Large frugivore	1	0.01 (1)
Cingulata						
Dasypodidae	*Dasypus kappleri*	In [28]	9.50 [24]	-	2	0.06 (5)
	*Dasypus novemcinctus*	In [28]	5.50 [28]	-	4	0.06 (5)
	*Priodontes maximus*	In [28]	38.00 [24]	-	1	0.01 (1)
Pilosa						
Myrmecophagidae	*Myrmecophaga tridactyla*	In [28]	22.33 [24]	-	3	0.03 (3)
Rodentia						
Cuniculidae	*Cuniculus paca*	Sp/Fr [28]	8.00 [28]	Large frugivore	6	0.31 (28)
Dasyproctidae	*Myoprocta acouchy*	Sp/Fr [54]	0.95 [24]	Small frugivore	9	0.41 (37)
	*Dasyprocta leporina*	Sp/Fr [54]	3.50 [24]	Medium frugivore	7	0.11 (10)
Sciuridae	*Sciurus aestuans*	Sp/Fr [28, 52]	0.19 [24]	Small frugivore	1	0.01 (1)

*Trophic guild: (Fr) frugivore, (Fo) folivore, (Sp) seed predator, (Fu) fungus, (In) Invertebrate predator, (Vp) terrestrial vertebrate predator, (Fp) fish predator.

**Average relative abundance (number of independent videos of each species divided by 900 camera-trap days and multiplied by 10 camera-trap days, rounded to two decimal points).

### Environmental variables

We used four environmental variables to explain the dry season distribution of the vertebrate groups and species (S1 Table). Fallen fruit availability was sampled in three plots (250 x 2 m) at each of the 30 permanent sampling points where camera traps were located: (1) the RAPELD permanent plot and (2) another two trail plots located 250 m before and after the location of the camera along the east-west trails. All fallen fruits within the plots were identified *in situ* using field guides [58, 59]. Identification of all fruits found in the field was also confirmed with the aid of a trained technician from the Amapá State Scientific Research and Technology Institute (Instituto de Pesquisas Científicas e Tecnológicas do Estado de Amapá—IEPA) and the Emilio Goeldi Museum of Pará State—MPEG. Only known animal-consumed fruit species were considered [14, 18, 60] (S2 Table). Finally, fruit availability was expressed as the presence or absence of fruits for each of the 30 sample points.

To evaluate the spatial distribution of water availability we used two variables at different scales: (1) distance from camera traps to the nearest large river and (2) distance from camera traps to the nearest stream with water. The distance from the camera traps to the nearest large river was estimated by using shapefiles of the Araguari and Falsino rivers (available at http://hidroweb.ana.gov.br/HidroWeb.asp?TocItem=4100), and measured as a straight line (Euclidian) distance with ArcGIS version 10.2 [61]. Distance from the camera traps to the nearest stream with water was estimated *in situ*, using a handheld Global Positioning System (GPS) while walking along the east-west trails in the RAPELD grid during the camera trapping survey period. The distance from each camera station to the nearest stream with water was calculated as a straight line (Euclidian) distance using ArcGIS version 10.2 [61].

To estimate the altitude of the terrain at the camera trap locations, we used a digital elevation model (DEM) produced by the Shuttle Radar Topographic Mission (SRTM) [62], with a 3 arc-second spatial resolution (approximately 90 m at the Equator), downloaded from http://www.cgiar-csi.org/data/srtm-90m-digital-elevation-database-v4-1. Altitude values for each camera trap were then obtained by overlaying camera trap coordinates with the SRTM DEM.

### Data analysis

The relative abundance of each species was expressed as the number of independent videos of each species divided by the sampling effort (900 camera trap-days) and multiplied by 10 trap-days (rounded to two decimal points), with which we were able to make comparisons with other studies of Amazon forest vertebrates [7, 46].

To assess whether the sampling effort was sufficient to record the majority of species, we constructed and compared cumulative species curves with function *specaccum* of the *Vegan* package [63]. To predict the total number of species that could potentially be detected in the study area, we used the First order jackknife estimator to extrapolate the species richness (i.e., estimate the number of undetected species) based on the frequency of recorded species (function *specpool*, package *Vegan*) [63].

We used Spearman correlation values to evaluate independence between environmental variables. Variables with weak correlations (r_s_ < 0.50) were retained for use in subsequent analyzes.

To test for differences in the ecological relationships of different functional groups and species we used Generalized Linear Models (GLMs, error distribution family = Tweedie) [64]. GLMs were preferred to alternatives such as occupancy models because the number of videos (i.e., potential of recaptures) and naïve occupancy (proportion of cameras with records) was low for most species. These low sample sizes meant that it was only possible to obtain reliable occupancy estimates for two of the seven most commonly recorded species (S1 File).

The GLMs were run separately for each species and for the five functional groups of vertebrates (Table 1). For the GLM analysis we selected only groups/species with at least one video in five or more different cameras within the study area. In addition to the additive effect of the four variables we also included the interaction between distance to nearest water and presence of fruits. To improve numerical stability of the GLMs and interpretation of the interaction term, the continuous variables were standardized (centered and scaled by the standard deviation). All analyses were performed with the *R* language and environment for statistical computing [65].

## Results

### Sampling effort and species richness

Following a sampling effort of 900 trap-days (30 days for each camera-trap), we obtained 423 videos of which 279 were considered independent of 25 vertebrate species (Table 1). We obtained an overall capture rate of 0.31 videos per trap-day (279 independent videos /900 trap-days). This total included four bird and 21 mammal species, representing 10 orders: Birds—Tinamiformes, Galliformes, Gruiformes; Mammals—Artiodactyla, Perissodactyla, Carnivora, Cingulata, Pilosa, Didelphimorphia and Rodentia (Table 1). From the 25 vertebrate species we detected a total of 16 frugivores (Table 1, S3 Table), including four bird and 12 mammal species (four rodents, four ungulates, three carnivores and one marsupial).

**Fig 2:**
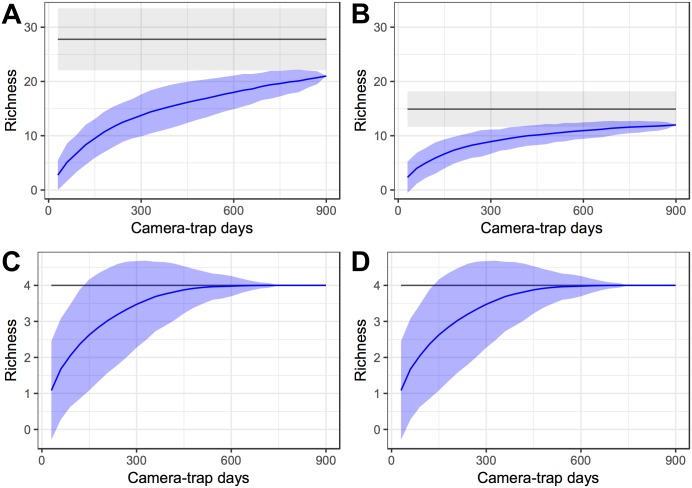
Cumulative curves for mammal and bird species sampled with camera traps in the dry season in the Amapá National Forest. Detection of species recorded in 900 camera-trap days randomized 1000 times and results used to derive mean (blue line) 95% confidence intervals of the mean (blue polygon). First order jackknife estimates of extrapolated species richness and 95% confidence intervals are showed with black line and light gray shaded area, respectively. (A) Cumulative curve for all mammal species; (B) Cumulative curve for frugivorous mammals; (C) Cumulative curve for all bird species; (D) Cumulative curve for frugivorous birds.

The species accumulation curves reached an asymptote for all birds, frugivorous mammals and frugivorous birds (Fig 2). Although the accumulation curve did not reach an asymptote for all mammals (Fig 2A), we obtained 75.63% of the expected mammal species. For all birds we obtained 100% of the species pool. This suggests that sampling effort was sufficient for frugivorous vertebrates.

### Functional groups

**Fig 3:**
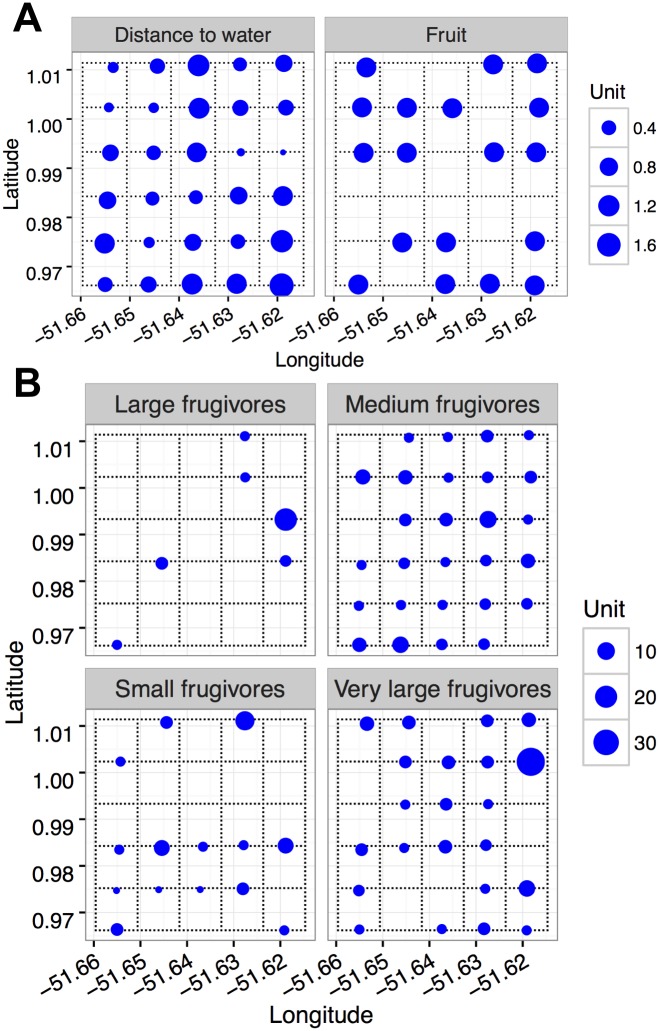
(A) Distance to the nearest water (km) and presence/absence of fruits and (B) number of photos of functional groups of frugivores per sampling point on a 25 km^2^ grid, during the dry season in the Amapá National Forest, Brazil.

As none of the four environmental variables were strongly correlated (Spearman r ranging between 0.00 and 0.26) we retained all in subsequent analyses. The explanatory power of the GLMs was low for almost all groups (Table 2), with a maximum deviance explained of 49% (for large frugivores) and a minimum of 10% (for small frugivorous). Two groups (all frugivores and small frugivores) were not significantly influenced by any of the environmental variables measured. The groups containing medium (1–5 kg) and large frugivores (5–15 kg) were negatively influenced by distance to nearest water. The groups of large and very large frugivores were significantly influenced by altitude. However, while large frugivores were negatively influenced by altitude, the very large frugivores were positively influenced by this variable. None of the groups showed a statistically significant association with the presence of fruits and only a marginally significant (*P* < 0.10) interaction between distance to the nearest water and presence of fruits was found for medium frugivores (Fig 3, Table 2).

**Table 2:** Parameter (Slope) estimates of explanatory variables from the GLMs on the abundance of groups of vertebrates in the eastern Brazilian Amazon.

	All frugivores^a^		Small frugivores^b^		Medium frugivores^c^		Large frugivores^d^		Very large frugivores^e^	
	Slope (SE)^f^	t value	Slope (SE)^f^	t value	Slope (SE)^f^	t value	Slope (SE)^f^	t value	Slope (SE)^f^	t value
Intercept	1.828 (0.751)	2.43*	0.871 (1.540)	0.57	-0.020 (0.624)	-0.03	-2.740 (2.039)	-1.34	1.390 (1.125)	1.24
Altitude	0.059 (0.166)	0.35	-0.097 (0.382)	-0.25	-0.233 (0.144)	-1.61	-1.145 (0.488)	-2.35*	0.665 (0.265)	2.51*
Distance to large rivers	0.119 (0.186)	0.64	0.255 (0.405)	0.63	-0.125 (0.132)	-0.95	-0.052 (0.378)	-0.14	0.243 (0.316)	0.77
Distance to nearest water	-0.301 (0.208)	-1.45	-0.486 (0.528)	-0.92	-0.481 (0.192)	-2.51*	-2.459 (0.906)	-2.72*	0.400 (0.294)	1.36
Fruit (presence vs absence)	0.321 (0.754)	0.43	-0.906 (1.530)	-0.59	0.727 (0.611)	1.19	-0.311 (1.756)	-0.18	-0.248 (1.171)	-0.21
Distance to nearest water X Fruit (presence vs absence)	0.209 (1.071)	0.20	-0.428 (2.343)	-0.18	1.649 (0.843)	1.96^†^	3.058 (3.189)	0.96	-1.420 (1.593)	-0.89
Model deviance explained (%)^g^	13.90		9.90		29.50		48.80		29.20	
Model AIC^h^	198.77		105.90		125.65*		71.88***		149.14^†^	

Significance values:

^†^p<0.10,

*p <0.05,

***p<0.001.

^a^ Includes relative abundances of all frugivores recorded in the study area;

^b^ Includes relative abundances of only small frugivores (< 1 kg);

^c^ Includes relative abundances of only medium frugivores (1–5 kg);

^d^ Includes relative abundances of only large frugivores (5–15 kg);

^e^ Includes relative abundances of only very large frugivores (> 15 kg);

^f^ Slope for variables and Standard Error (SE);

^g^ Percentage of Deviance Explained for each model (%);

^h^ Akaike Information Criterion value for each model (AIC);

### Species

*Psophia crepitans* was the most frequently recorded species with 55 records (0.61 records/10 trap-days), followed by *Pecari tajacu* with 44 records (0.49 records/10 trap-days), *Myoprocta acouchy* with 37 records (0.41 records/10 trap-days) and *Mazama americana* with 32 records (0.36 records/10 trap-days) (Table 1). Of the seven most common species assessed in the GLMs, three showed statistically significant results (Table 3). The percentage of variation explained by the models ranged from a minimum of 6% for *Mazama nemorivaga* to a maximum of 48% for *Cuniculus paca*. Of these seven species, the rodent *C*. *paca*, the ungulate *M*. *americana* and the rodent *D*. *leporina* were the species where the model provided the highest percentage of explanation for their relative abundances, ranging from 36 to 48% (Table 3).

**Table 3:** Parameter (Slope) estimates of explanatory variables from the GLMs on the abundance of most common species of vertebrates in the eastern Brazilian Amazon.

	*Cuniculus paca*		*Dasyprocta leporina*		*Mazama americana*		*Mazama nemorivaga*		*Myoprocta acouchy*		*Pecari tajacu*		*Psophia crepitans*	
	Slope (SE)^a^	t value	Slope (SE)^a^	t value	Slope (SE)^a^	t value	Slope (SE)^a^	t value	Slope (SE)^a^	t value	Slope (SE)^a^	t value	Slope (SE)^a^	t value
Intercept	-2.709 (2.031)	-1.33	-1.745 (1.551)	-1.13	-1.612 (1.418)	-1.14	-2.098 (1.910)	-1.10	0.244 (1.633)	0.15	1.915 (1.458)	1.31	-0.566 (0.892)	-0.64
Altitude	-1.134 (0.487)	-2.33*	-0.164 (0.420)	-0.39	1.657 (0.380)	4.37***	0.707 (0.431)	1.64	-0.017 (0.394)	-0.04	0.286 (0.300)	0.95	-0.133 (0.194)	-0.69
Distance to large rivers	-0.053 (0.378)	-0.14	0.255 (0.343)	0.74	0.171 (0.470)	0.36	-0.066 (0.492)	-0.14	0.242 (0.426)	0.57	0.141 (0.379)	0.37	-0.345 (0.181)	-1.90^†^
Distance to nearest water	-2.432 (0.900)	-2.70*	0.320 (0.575)	0.56	0.220 (0.454)	0.49	0.188 (0.446)	0.42	-0.434 (0.558)	-0.78	0.251 (0.312)	0.80	-0.924 (0.336)	-2.75*
Fruit (presence vs absence)	-0.331 (1.753)	-0.19	-0.461 (1.795)	-0.26	0.551 (1.404)	0.39	1.449 (1.932)	0.75	-0.335 (1.614)	-0.21	-1.343 (1.500)	-0.90	0.566 (0.811)	0.70
Distance to nearest water X Fruit (presence vs absence)	3.013 (3.173)	0.95	1.324 (1.979)	0.67	0.309 (1.891)	0.16	1.032 (2.302)	0.45	0.185 (2.412)	0.08	-3.555 (2.518)	-1.41	1.904 (1.297)	1.47
DE (%)^b^	48.10		36.00		45.60		6.20		21.10		16.30		36.20	
Model AIC^c^	71.76***		58.92^†^		82.05***		91.51		88.31		106.88		108.86**	

Significance values:

^†^p<0.10,

*p <0.05,

**p<0.01,

***p<0.001.

^a^ Slope for variables and Standard Error (SE);

^b^ Percentage of Deviance Explained for each model (DE(%));

^c^ Akaike Information Criterion value for each model (AIC);

*Cuniculus paca* and *P*. *crepitans* were the only species with two marginally or significant variables in the GLMs. *M*. *americana* was significantly associated with one variable in the model, while *D*. *leporina*, *M*. *nemorivaga*, *P*. *tajacu* and *M*. *acouchy* were not associated significantly with any of the environmental variables (Table 3).

The variable altitude had a positive influence on the relative abundance of one ungulate (*M*. *americana)* and negative influence for *C*. *paca*. The distance to large river had a marginal negative influence only for the bird *P*. *crepitans*. Finally, distance to nearest water had a negative effect on *C*. *paca* and *P*. *crepitans*.

## Discussion

Our study showed that water availability is more important than the presence of fallen fruit during an event of resource scarcity in a lowland Amazon forest. Thus, our findings support the prediction that water availability would affect functional groups and species of terrestrial frugivores. In contrast we found no support for the prediction that fallen fruit would also affect meso-scale patterns in terrestrial frugivores. Distance to nearest water was an important variable that explained meso-scale variation in two functional groups (medium and large frugivores) and two terrestrial frugivore species. These observations allow an improved understanding of species-specific responses to water scarcity, and also provide baseline information for monitoring tropical frugivore responses to future environmental changes. However, we must remain cautious in our conclusions as we only surveyed a single dry season and our results should be interpreted carefully.

### Sampling effort and species richness

The difference between the observed and extrapolated species richness values obtained indicates that our sampling effort was sufficient to record the full spectrum of frugivorous birds and mammals in the study area. Indeed, the 25 species recorded in our study is similar to the composition and number of species recorded for other Amazonian regions [66–68]. A previous camera-trap survey in the same study area reported a total richness of 25 species with a sampling effort of 1800 trap-days (900 trap-days each for the dry and rainy seasons), with 21 vertebrate species recorded during the 2013 dry season [7]. Based on these findings we consider the results from the present study to be representative of the terrestrial vertebrate species in the study area.

For mammals, the species compositions of the two studies during the dry season were similar, with a difference that *Tamandua tetradactyla* was detected only in 2013 [7] and *Priodontes maximus* was detected only in 2015. We also detected four species that were not recorded in a previous study [7] during the dry season but were registered in the rainy season: *Sciurus aestuans*, *Procyon cancrivorus*, *Nasua nasua* and *Speothos venaticus*. Such differences are consistent with the findings from previous studies that show spatial and temporal variations in the species recorded using camera-trap surveys in tropical forests [22, 66, 69, 70].

Four mid-sized and large-bodied terrestrial bird species were recorded in our study. Due to their body sizes and habit of foraging on the ground [6, 55], these birds are likely to be recorded by camera trap studies [7, 24]. The same bird species were previously recorded during the 2013 dry season in the study area, with similar relative abundances to our study [7]. Although our extrapolated bird richness values suggest that we registered all the bird species that could possibly be recorded with camera traps, the frequency with which they were registered was generally low. This result may suggest that camera traps might not be ideal for monitoring this group of birds, and that complementary techniques maybe necessary. Indeed, a combination of indirect and direct techniques have been proven to be more efficient than cameras traps only [71].

Camera trapping studies are often conducted during the dry season in tropical forests. For example, dry season surveys form the basis of the camera-trap protocols implemented globally by the Tropical Ecology Assessment and Monitoring Network [70, 72]. This preference for dry season surveys is mainly due to the logistical constraints associated with rainy season surveys (e.g. restricted access and cameras malfunctioning). A previous study in our study area [7] found fewer records of some frugivore species during the dry compared with the rainy season. In our study, several vertebrates were represented by only a few photographs, showing the difficulty of detecting some frugivorous during the dry season (e.g., *Tapirus terrestris*, *Crax alector*, *Crypturellus erythropus* and *Tinamus major*).

### Differences between functional groups

The completeness of our survey enabled us to examine if different resources (fruit and water) explained the relative abundance of a broad spectrum of terrestrial frugivores. For example, our functional body sizes ranged over three orders of magnitude (<1 to > 100 kg). All our functional groups include species that are ubiquitous across tropical and sub-tropical regions of South America [52, 53]. As such we can expect a wide range of physiological and behavioral adaptations and acclimations to seasonal changes in resources both within and between groups.

Our results agree with previous studies that found other factors to be more important than fruit availability to explain variation in the abundance of terrestrial frugivores [1, 2, 4, 73]. Medium frugivores were influenced only by distance to the nearest water. Large frugivores were most strongly influenced by distance to nearest water and altitude, while very large frugivores were only influenced by altitude. Medium and large frugivores were associated negatively with distance to nearest water showing a significant increase in the number of records in areas closer to water bodies within the study area. This finding supports previous studies that show water availability may play a stronger role in driving the behavior of large bodied terrestrial mammals than food searching during the dry season [1–4].

The group of medium to large-bodied vertebrates is one of the most affected by subsistence hunting [18, 26] and it has been shown that in semi-arid regions these vertebrates can be more easily hunted closer to water during the dry season [74]. Typically hunter behavior does not seem to show great variation in the neotropics and regional differences are more related to the use of certain species (notably primates) by native Americans and European descendants [75]. Although the rivers are the main means of transport of habitants in the Amazon region [26], we did not find any negative effect on the relative abundance of these frugivorous groups close to the large rivers, which supports the idea that there is currently little anthropogenic impact within the ANF [7].

Altitude was the best predictor of the relative abundances of the large and very large-bodied frugivores. However, while large frugivores were negatively associated with altitude, very large frugivores were positively associated with the same variable. A similar pattern was observed in some large-bodied frugivores that remained in the highlands during the rainy season but moved to lowlands in the dry season, potentially responding to the renewed supply of resources following the rainy season [11, 76]. SRTM altitude varies from sea level to 1216 m across the >5 million km^2^ of Brazilian Amazonia. However, the mean altitude is 159.5 m, and there is low meso-scale variation (SD values < = 40) in 90% of the area [40]. Nevertheless, even with this low variation, altitude is a key determinant of Amazonian biodiversity [31–34], affecting soil, water availability, climate and other biotic and abiotic variables [30].

We found only weak associations between the sampled community of terrestrial frugivores and the meso-scale availability of fallen fruits. A lack of association of the interaction between distance to the nearest water and presence of fruits was also confirmed for all groups, with the exception of medium frugivores that were weakly associated with this interaction. The simplest and most likely explanation for this lack of association is the generalist nature of the frugivore species. All species consume fruits but all may also consume a variety of alternative foods [6, 10, 52, 53, 77, 78]. As such, our findings support the idea that terrestrial fruit-frugivore relationships tend to be less strong and less affected by fruit availability compared with canopy fruit-frugivore relationships [9, 17, 18, 79].

Studies from other Amazon terra firme forests can help to understand our observations of the groups of small to large frugivores in the ANF. Previous studies suggest that species dietary diversity and ability to adapt to a changing resource base are important traits in determining vertebrate responses to relative food reduction within terra firme forests [25]. It has also been shown that fruit–frugivore networks are also highly diffuse in this biome [11]. The interaction between medium to large-bodied frugivores and fruit resources suggests generalization in terra firme forests, compared with greater specialization in varzea forests for this group [11]. One driver of generalization in fruit–frugivore relationships could be dietary complementarity [79]. Diets of the studied groups are rarely entirely frugivorous, with fruit consumed in varying ratios depending on species-specific interactions with habitat, season, fruit availability and the availability of alternative food sources [17, 18, 78, 80]. Thus, for small frugivorous mammals, there is evidence that leaf and fiber consumption increases during periods of fruit scarcity [78]. It has also been suggested that small-bodied species are less likely to be affected by habitat changes because they may be able to diverge through microhabitat specialization [25], supporting our results in this body-sized group. Large and very-large frugivores are also not strictly dependent on fruits, for example, deer and tapir form part of the browser-grazer community and have the ability to digest leaves [81, 82]. Previous studies show that within the very-large frugivores, digestive physiology is more important than body size for resource portioning of diet [77], with all species consuming a mix of fruit, leaves and fiber that varies with habitat and season [77, 81, 82]. Thus, a weak relationship between fruit availability and these functional groups is not surprising.

### Differences between species

Our findings support those from previous studies, which found a greater number of detections of *Cuniculus paca* in low-lying areas near permanent water sources [7]. On the other hand, *Mazama americana* showed a greater number of detections within the study area uplands. These observations support the importance of altitude as a driver and modulator of species meso-scale distribution patterns [40]. Similarly, a previous study [7] found the same association in relation to streams for both *Mazama americana* and *Pecari tajacu* within the ANF. These results contrast to those from other studies in more arid regions, which found that the dry season distributions of some ungulates were strongly influenced by water availability [1–4]. Keuroghlian, Eaton [83] reported seasonal movements of some ungulates apparently driven by availability of key fruits in a tropical forest. However, dry season fruit availability was not a significant variable for ungulates in our study.

Only one bird species (*P*. *crepitans*) had sufficient records for analysis. Fruit and seeds are known to form the bulk of the diet in *P*. *crepitans*, *C*. *alector* and *T*. *major*, however, it is known that *P*. *crepitans* also eats invertebrates in relatively large quantities [6]. We found that during the dry season, the presence of fruit had no significant effect on the relative abundance of *P*. *crepitans*. Chatterjee and Basu [73] suggest that other factors such as insect abundance may be important for frugivore bird groups that also rely on insects as a secondary dietary component. For these authors, a combination of fruit availability and insect availability should explain the variation in frugivore bird density in space and time, rather than fruit availability alone. Although our observations might support this conclusion, *C*. *alector* and *T*. *major* had few records (videos in < 5 different camera traps) and the relationship with fruit availability could not be evaluated. However, our study supports findings that *P*. *crepitans* has a preference for areas close to water availability and close to large rivers within Amazonian forests [6, 7].

Although camera traps are efficient for rapid inventories during the dry season [84], we must remain cautious in our conclusions. Capture frequencies with camera traps can give an idea of the relative abundance of different species, but may be affected by a variety of factors such as species-specific behavior (e.g. use or avoidance of trails, partly arboreal versus exclusively terrestrial, or habitat specialist versus generalist) [66, 69]. For this reason we limit our conclusions to differences in spatial encounter rates and do not attempt to imply population parameters (e.g. density).

## Conclusions

Our models could only partially explain dry season abundance patterns in the recorded species and groups. The lack of association between frugivores and fruit availability could suggest that, at the meso-scale level (25 km^2^), other factors may have more decisive roles during a period of resource scarcity. We found that, at this scale, the distribution of frugivore species and functional groups can be partly explained by variables such as water availability and altitude. However, a substantial survey effort is necessary to ensure a representative sample of terrestrial frugivores and to better understand the processes driving the spatial distribution of these vertebrate groups.

## Supporting information

S1 File Results from the occupancy models. (DOC)

S1 Fig Monthly rainfall recorded close (36 km) to the Amapá National Forest study site. Weather station data available from the Brazilian National Water Agency (station ID: 8052000). Monthly totals are presented from three years (2013, 2014 and 2015). Boxplots show means and 95% confidence limits estimated via nonparametric bootstrap. The blue line and shaded areas are the mean value and 95% confidence intervals from a GAM model illustrating the trend in rainfall.
(DOC)

S1 Table Explanatory variables obtained during the dry season (October-December 2015) in the Amapá National Forest, eastern Brazilian Amazon. (DOC)

S2 Table List of fruits identified during the dry season (October-December 2015) in the Amapá National Forest, eastern Brazilian Amazon. (DOC)

S3 Table Number of independent captures obtained by camera trapping for all frugivores during the dry season (October-December 2015) in the Amapá National Forest, eastern Brazilian Amazon. (DOC)
