# Supplementary material for: Water availability not fruitfall modulates the dry season distribution of frugivorous terrestrial vertebrates in a lowland Amazon forest
Source: PLoS One. 2017 Mar 16;12(3):e0174049. doi: 10.1371/journal.pone.0174049 (PMC5354462; doi:10.1371/journal.pone.0174049)
Supplement: S1 File — (DOC) [file pone.0174049.s001.doc]

**Water availability not fruitfall modulates the dry season distribution of frugivorous terrestrial vertebrates in a lowland Amazon forest**

Omar Stalin Landázuri Paredes, Darren Norris, Tadeu Gomes de Oliveira, Fernanda Michalski

**S1 File. Results from the occupancy models.**

**Introduction**

Occupancy analysis can enable the robust estimation of community and population parameters from camera-trap data (Burton et al., 2015). The estimation and interpretation of occupancy model parameters and associated biological inference depends on meeting model assumptions (Bailey et al., 2014; Burton et al., 2015; O’Connell and Bailey, 2011), yet camera trap surveys frequently fail to meet all or several of these assumptions (Bailey et al., 2007; Burton et al., 2015). As such, a number of decisions are made post-sampling when modelling occupancy using camera-trap data. These decisions can have strong influences on model results (Burton et al., 2015). For example, data from remote cameras is continuous; therefore the first decision is how to discretize camera data to create capture histories suitable for occupancy analysis (Burton et al., 2015; Hines et al., 2010). Despite its importance there is as yet little understanding as to how different capture histories (i.e. choice of sampling replicate length) may affect camera trap occupancy estimates. To understand whether it would be possible to apply occupancy modelling techniques to quantify meso-scale distribution and abundance we analyzed data from the videos of the seven most commonly detected species in the Amapá National Forest (ANF). We firstly asked whether occupancy models could be estimated for all seven species using the same covariates as our GLMs, we then asked to what extent imperfect detection influenced occupancy modelling for our study species.

**Materials and methods**

Occupancy of the seven most frequently detected species was modelled using the maximum likelihood approach implemented in the R package unmarked version 0.11-0 (Fiske and Chandler, 2011). We discretized the 30 days of data for each species into different replicate lengths (2, 3, 5, 6, 10 and 15 days). As a result, the number of sample replicates (k) varied from 15 – 2 replicates, respectively. For each discretization of the same data, the single season occupancy (MacKenzie et al., 2002) was estimated with the “occu” function. As we were interested in knowing whether it would be possible to use occupancy models to address study objectives, a total of five models (Table S1) were estimated for each discretization. These five models included different covariates (Table S1) that were generally the most important covariates in our GLM analysis.

**Table S1. Models used to estimate occupancy. A total of 5 models were used to estimate occupancy of seven most common species in the Amapá National Forest (ANF). “Minimum” models included the two most generally important covariates according to the GLM results. “Full” models included five covariates as described in the full article.**

| Model | State covariates^a^ | Detection covariates |
| --- | --- | --- |
| Null | ~1 | ~1 |
| Minimum detection | Altitude + Distance to nearest water | Event |
| Minimum no detection | Altitude + Distance to nearest water | ~1 |
| Full detection | Altitude + Distance to nearest water + Distance to large rivers + Presence fruit + Distance to nearest water X Fruit | Event |
| Full no detection | Altitude + Distance to nearest water + Distance to large rivers + Presence fruit + Distance to nearest water X Fruit | ~1 |

^a^ The same as used in GLM models

To determine if the models of our data could be useful we used a two stage approach. Firstly, we checked that models could be built and estimated. Model building and estimation in unmarked has been developed as a general solution to a wide variety of use cases (Fiske and Chandler, 2011). To meet needs of multiple uses, unmarked includes various data and model checking steps that are revealed to users via error and warning messages. When it was not possible to generate numerical estimates using the function default settings, we followed guidance from online user groups (e.g. [https://groups.google.com/forum/#!forum/unmarked](https://groups.google.com/forum/#!forum/unmarked)) to correct errors/warnings via manually adjusting model fitting parameters (e.g. start values, number of iterations for convergence). Secondly we checked the adequacy of model fit using a parametric bootstrap of the χ^2^ statistic appropriate for binary data (Fiske and Chandler, 2015).

Occupancy estimation models imperfect detectability via repeat sampling occasions. In our case to represent the imperfect detection of camera-traps we discretized the continuous 30 day survey period of each camera trap to generate detection histories of different replicate lengths (2, 3, 5, 6, 10 and 15 days). To determine the importance of imperfect detection on species occupancy we firstly compared models with and without detection covariates. We compared the AIC values from minimum models, both with two state covariates (Table S1) with and without detection covariates. These minimum models were chosen as numerical estimation was not possible with more covariates across the different replicate lengths (2, 3, 5, 6, 10 and 15 days). If detection covariates improve the model in the paired comparison, we would expect delta AIC values to be 0 for the detection model. We then compared estimated and naïve occupancy values. The proportion of sites occupied was calculated using empirical Bayes methods (Fiske and Chandler, 2015). This estimate applies to the hypothetical population of all possible sites, not the sites found in our sample. If occupancy modelling provided improvements over naïve occupancy (in other words corrected biases in the naïve values) we would expect that the estimated 90% confidence intervals would not overlap our naïve values.

**Results and Discussion**

Based on our two stage evaluation of numerical estimation and model fit it was possible to estimate single season occupancy for the two most commonly detected species (*Psophia crepitans* and *Pecari tajacu*). A combination of numerical errors and poor model fit meant that it was not possible to reliably estimate occupancy for the other five species when including detection covariates.

Including detection history as a detection covariate did not generally improve the occupancy models as shown by the positive delta AIC values (Fig. S1). This suggests that imperfect detection is unlikely to have a strong influence over our 30 day single season survey period. The estimated occupancy values for *Pecari tajacu* overlapped naïve values but varied widely (90% confidence interval of the posterior ranging from 0.47 to 1.0, Fig. S1). This finding suggests that although model fit was adequate there remains considerable uncertainty in the occupancy model estimates for this species. There was less variation in the estimated occupancy for the more frequently recorded *Psophia crepitans* (Fig. S1), with estimates overlapping naïve values in 3 of the 6 replicate lengths (3, 6 and 15 days, Fig. S1). This supports previous studies that show the importance of both a relatively high naïve detection probability and post-sampling modelling decisions to obtain reliable occupancy estimates.

**Conclusion**

Our camera trap survey enabled us to model the single season occupancy of two species with occupancy and detection covariates. This finding agrees with previous studies that show surveys typically need both a large number of sites and species detection probability values above 0.8 to obtain reliable occupancy estimates (Long et al., 2008; MacKenzie and Royle, 2005).

|  | Goodness of Fit | Detection selection | Estimated occupancy |
| --- | --- | --- | --- |
| *Psophia crepitans* (21 of 30 sites with at least one vídeo) | 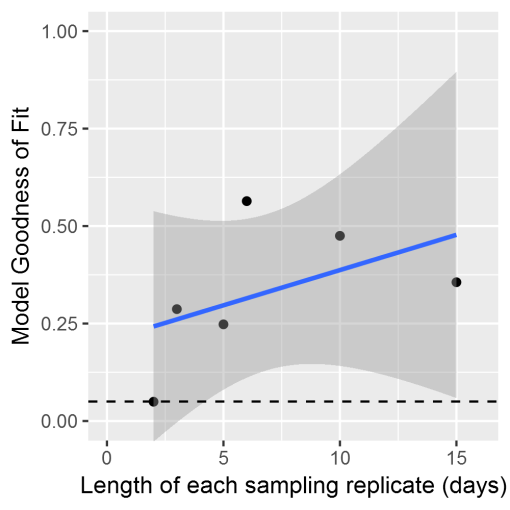 | 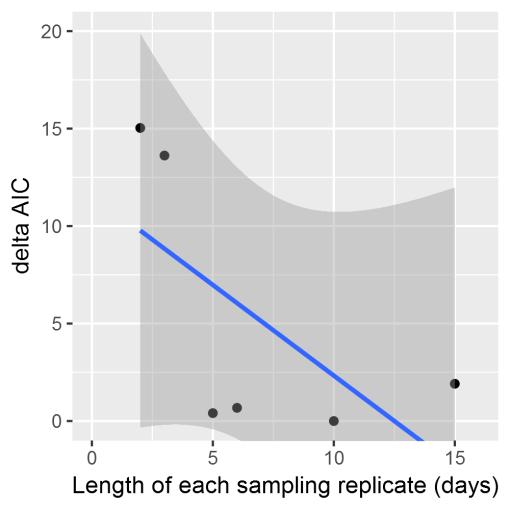 | 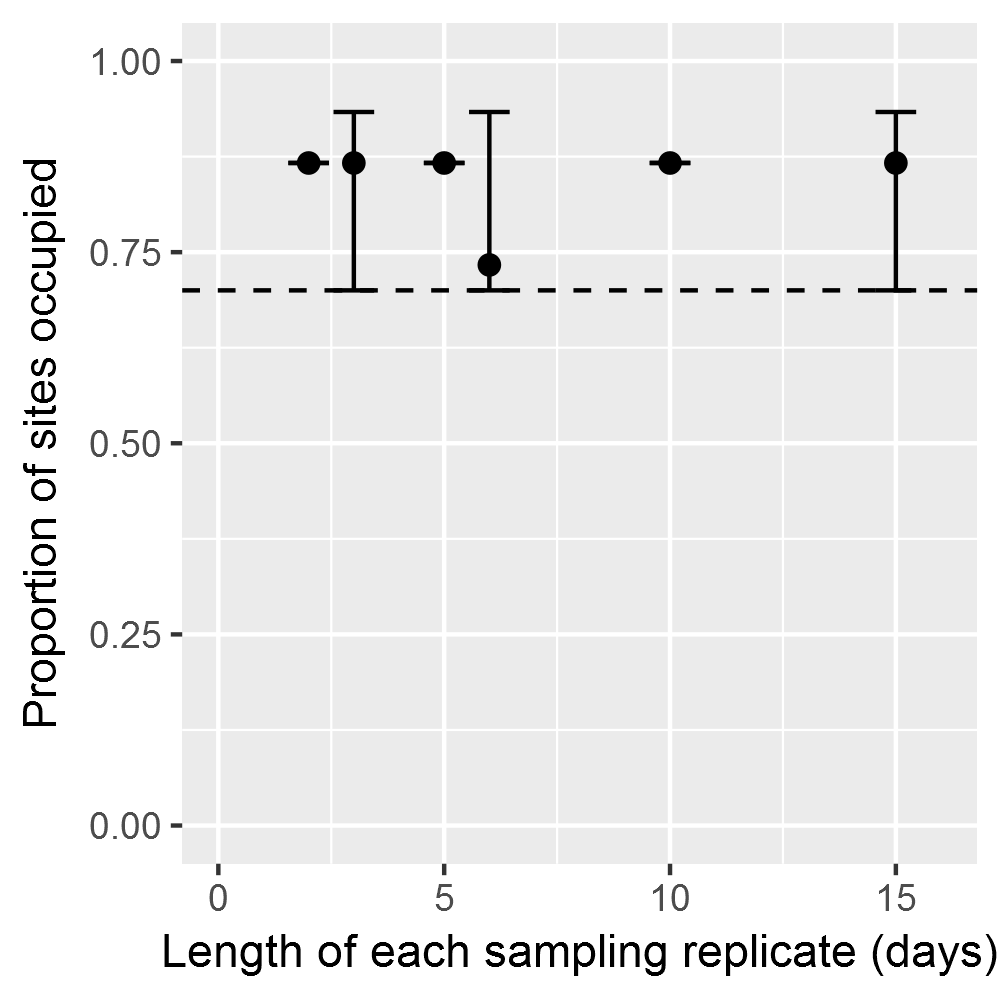 |
|  |  |  |  |
| *Pecari tajacu* (14 of 30 sites with at least one vídeo) | 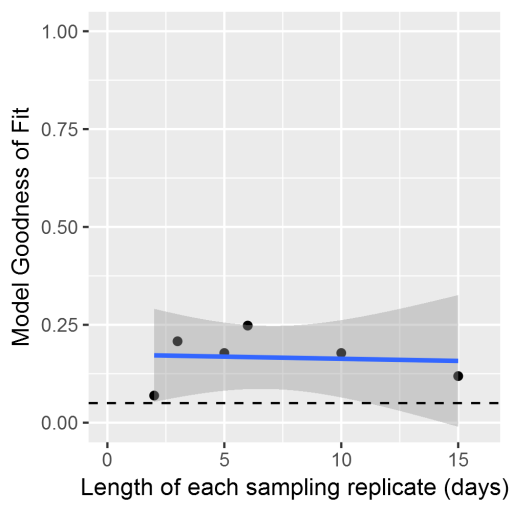 | 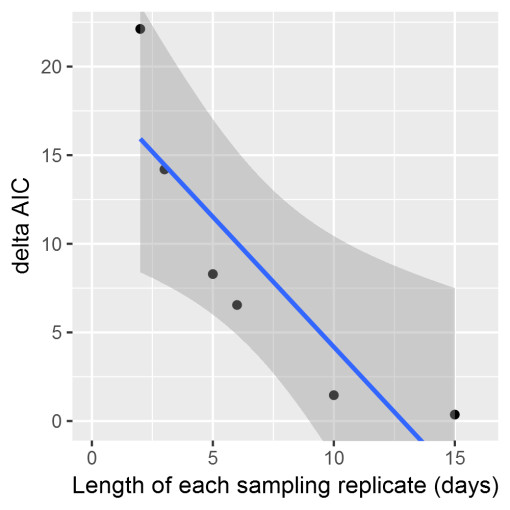 | 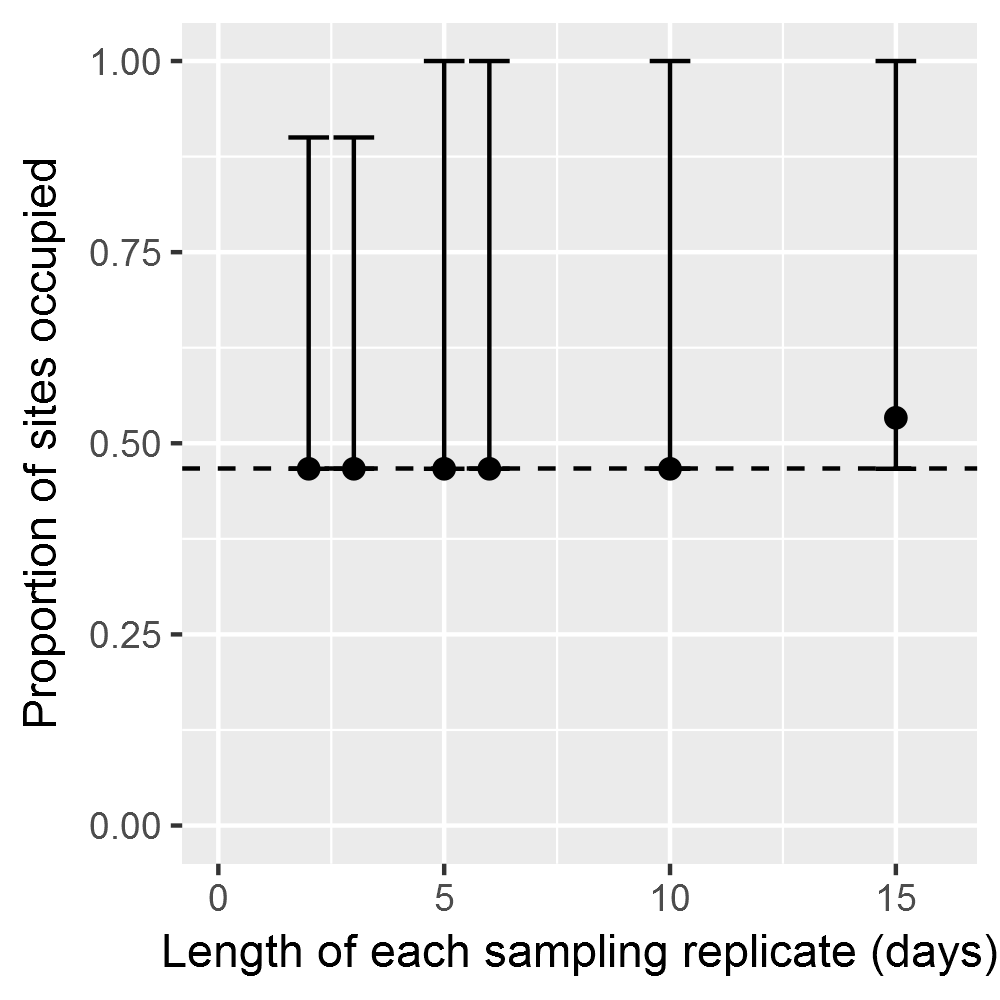 |

**Figure S1. Single season occupancy modelling of the two most frequently detected species. Graphs show comparison of model goodness of fit, selection of detection covariate model and estimated occupancy across different lengths of sampling replicate (2 – 15 days).**

References

Bailey, L.L., Hines, J.E., Nichols, J.D., MacKenzie, D.I., 2007. Sampling design trade-offs in occupancy studies with imperfect detection: examples and software. Ecological Applications 17, 281-290.

Bailey, L.L., MacKenzie, D.I., Nichols, J.D., 2014. Advances and applications of occupancy models. Methods in Ecology and Evolution 5, 1269-1279.

Burton, A.C., Neilson, E., Moreira, D., Ladle, A., Steenweg, R., Fisher, J.T., Bayne, E., Boutin, S., 2015. REVIEW: Wildlife camera trapping: a review and recommendations for linking surveys to ecological processes. Journal of Applied Ecology 52, 675-685.

Fiske, I., Chandler, R., 2011. unmarked: An R package for fitting hierarchical models of wildlife occurrence and abundance. Journal of Statistical Software 43, 1-23.

Fiske, I., Chandler, R., 2015. Overview of unmarked: an R package for the analysis of data from unmarked animals.

Hines, J., Nichols, J., Royle, J., MacKenzie, D., Gopalaswamy, A., Kumar, N., Karanth, K., 2010. Tigers on trails: occupancy modeling for cluster sampling. Ecological Applications 20, 1456-1466.

Long, R.A., Zielinski, W.J., Long, R., MacKay, P., Zielinski, W., Ray, J., 2008. Designing effective noninvasive carnivore surveys. Noninvasive survey methods for carnivores, 8-44.

MacKenzie, D.I., Nichols, J.D., Lachman, G.B., Droege, S., Andrew Royle, J., Langtimm, C.A., 2002. Estimating site occupancy rates when detection probabilities are less than one. Ecology 83, 2248-2255.

MacKenzie, D.I., Royle, J.A., 2005. Designing occupancy studies: general advice and allocating survey effort. Journal of Applied Ecology 42, 1105-1114.

O’Connell, A.F., Bailey, L.L., 2011. Inference for occupancy and occupancy dynamics, Camera traps in animal ecology. Springer, pp. 191-204.
