# Supplementary material for: Water availability not fruitfall modulates the dry season distribution of frugivorous terrestrial vertebrates in a lowland Amazon forest
Source: PLoS One. 2017 Mar 16;12(3):e0174049. doi: 10.1371/journal.pone.0174049 (PMC5354462; doi:10.1371/journal.pone.0174049)
Supplement: S1 Fig — Weather station data available from the Brazilian National Water Agency (station ID: 8052000). Monthly totals are presented from three years (2013, 2014 and 2015). Boxplots show means and 95% confidence limits estimated via nonparametric bootstrap. The blue line and shaded areas are the mean value and 95% confidence intervals from a GAM model illustrating the trend in rainfall. (DOC) [file pone.0174049.s002.doc]

**Water availability not fruitfall modulates the dry season distribution of frugivorous terrestrial vertebrates in a lowland Amazon forest**

Omar Stalin Landázuri Paredes, Darren Norris, Tadeu Gomes de Oliveira, Fernanda Michalski

Camera traps were installed and operational across the dry season (October – December 2015, S1 Figure). During the survey period total monthly rainfall was 22.4, 12.8 and 88.9 mm (monthly totals for October, November and December respectively).


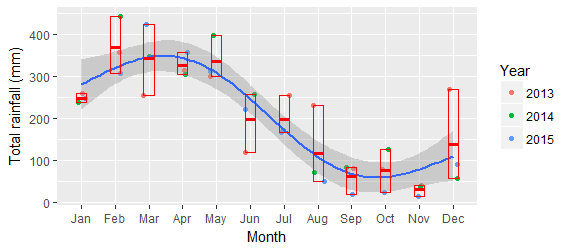


**S1 Figure. Monthly rainfall recorded close (36 km) to the Amapá National Forest study site. Weather station data available from the Brazilian National Water Agency (station ID: 8052000). Monthly totals are presented from three years (2013, 2014 and 2015). Boxplots show means and 95% confidence limits estimated via nonparametric bootstrap. The blue line and shaded areas are the mean value and 95% confidence intervals from a GAM model illustrating the trend in rainfall.**
