# Supplementary material for: Water availability not fruitfall modulates the dry season distribution of frugivorous terrestrial vertebrates in a lowland Amazon forest
Source: PLoS One. 2017 Mar 16;12(3):e0174049. doi: 10.1371/journal.pone.0174049 (PMC5354462; doi:10.1371/journal.pone.0174049)
Supplement: S1 Table — (DOC) [file pone.0174049.s003.doc]

**Water availability not fruitfall modulates the dry season distribution of frugivorous terrestrial vertebrates in a lowland Amazon forest**

Omar Stalin Landázuri Paredes, Darren Norris, Tadeu Gomes de Oliveira, Fernanda Michalski

**S1 Table. Exploratory variables obtained during the dry season (October-December 2015) in the Amapá National Forest, eastern Brazilian Amazon.**

Exploratory variables (A – D) per sampling point on a 25 km^2^ grid obtained during the dry season in the Amapá National Forest, Brazil.

| Site | A* | B* | C* | D* |
| --- | --- | --- | --- | --- |
| 1 | 110.44 | 2.19 | 1.68 | 1 |
| 2 | 107.58 | 2.23 | 1.04 | 1 |
| 3 | 123.89 | 1.90 | 1.14 | 1 |
| 4 | 119.17 | 1.13 | 0.53 | 0 |
| 5 | 117.92 | 0.76 | 0.43 | 1 |
| 6 | 100.36 | 2.70 | 1.38 | 1 |
| 7 | 120.39 | 3.17 | 0.40 | 0 |
| 8 | 127.94 | 2.50 | 0.63 | 1 |
| 9 | 105.22 | 1.96 | 0.15 | 1 |
| 10 | 115.72 | 1.37 | 1.05 | 0 |
| 11 | 110.53 | 3.16 | 0.99 | 0 |
| 12 | 110.19 | 3.97 | 0.71 | 0 |
| 13 | 124.61 | 3.20 | 0.33 | 0 |
| 14 | 124.58 | 2.24 | 0.34 | 0 |
| 15 | 116.67 | 1.23 | 0.70 | 0 |
| 16 | 101.64 | 2.85 | 0.01 | 1 |
| 17 | 101.53 | 3.79 | 0.03 | 1 |
| 18 | 115.50 | 4.79 | 0.98 | 0 |
| 19 | 129.44 | 2.25 | 0.39 | 1 |
| 20 | 118.11 | 1.35 | 0.60 | 1 |
| 21 | 140.31 | 2.63 | 0.50 | 1 |
| 22 | 112.69 | 3.66 | 0.54 | 0 |
| 23 | 136.25 | 3.56 | 1.13 | 1 |
| 24 | 140.58 | 2.65 | 0.11 | 1 |
| 25 | 104.53 | 1.78 | 0.09 | 1 |
| 26 | 132.06 | 2.81 | 0.67 | 1 |
| 27 | 124.72 | 3.76 | 0.33 | 1 |
| 28 | 134.06 | 4.16 | 1.28 | 0 |
| 29 | 148.39 | 3.15 | 0.46 | 0 |
| 30 | 132.53 | 2.30 | 0.14 | 1 |

* Variable names correspond to: A – Altitude (m), B – Distance to large rivers (km), C – Distance to nearest water (km), D – Fruit (presence vs absence).
