# Supplementary material for: Water availability not fruitfall modulates the dry season distribution of frugivorous terrestrial vertebrates in a lowland Amazon forest
Source: PLoS One. 2017 Mar 16;12(3):e0174049. doi: 10.1371/journal.pone.0174049 (PMC5354462; doi:10.1371/journal.pone.0174049)
Supplement: S2 Table — (DOC) [file pone.0174049.s004.doc]

**Water availability not fruitfall modulates the dry season distribution of frugivorous terrestrial vertebrates in a lowland Amazon forest**

Omar Stalin Landázuri Paredes, Darren Norris, Tadeu Gomes de Oliveira, Fernanda Michalski

**S2 Table. List of fruits identified during the dry season (October-December 2015) in the Amapá National Forest, eastern Brazilian Amazon.**

List of 12 families and 18 species of fruits identified in all sampling points on a 25 km^2^ grid during the dry season in the Amapá National Forest, Brazil.

| Type | Family | Species |
| --- | --- | --- |
| Fruit | Annonaceae | *Guateria* sp. |
|  | Arecaceae | *Astrocaryum* sp. |
|  |  | *Euterpe oleracea* |
|  |  | *Oenocarpus bacaba* |
|  | Caryocaraceae | *Caryocar villosum* |
|  | Chrysobalanaceae | *Licania* sp. |
|  | Clusiaceae | *Clusia grandiflora* |
|  | Fabaceae | *Inga* sp. |
|  |  | *Parkia pendula* |
|  |  | *Vatairea guianensis* |
|  |  | *Vouacapoua americana* |
|  | Lecythidaceae | *Eschweilera* sp. |
|  |  | *Gustavia augusta* |
|  | Malvaceae | *Theobroma subincanum* |
|  | Melastomataceae | *Bellucia grossularioides* |
|  | Meliaceae | *Carapa guianensis* |
|  | Myristicaceae | *Virola surinamensis* |
|  | Sapotaceae | *Chrysophyllum durifructum* |
