# Supplementary material for: Water availability not fruitfall modulates the dry season distribution of frugivorous terrestrial vertebrates in a lowland Amazon forest
Source: PLoS One. 2017 Mar 16;12(3):e0174049. doi: 10.1371/journal.pone.0174049 (PMC5354462; doi:10.1371/journal.pone.0174049)
Supplement: S3 Table — (DOC) [file pone.0174049.s005.doc]

**Water availability not fruitfall modulates the dry season distribution of frugivorous terrestrial vertebrates in a lowland Amazon forest**

Omar Stalin Landázuri Paredes, Darren Norris, Tadeu Gomes de Oliveira, Fernanda Michalski

**S3 Table. Number of independent captures obtained by camera trapping for all frugivores during the dry season (October-December 2015) in the Amapá National Forest, eastern Brazilian Amazon.**

Number of independent captures obtained by camera trapping for 16 species of frugivorous (A-P) per sampling point on a 25 km^2^ grid during the dry season in the Amapá National Forest, Brazil.

| Site | A* | B* | C* | D* | E* | F* | G* | H* | I* | J* | K* | L* | M* | N* | O* | P* |
| --- | --- | --- | --- | --- | --- | --- | --- | --- | --- | --- | --- | --- | --- | --- | --- | --- |
| 1 | 0 | 0 | 0 | 0 | 0 | 0 | 0 | 1 | 0 | 0 | 0 | 0 | 0 | 1 | 0 | 0 |
| 2 | 0 | 0 | 0 | 0 | 1 | 0 | 0 | 3 | 0 | 0 | 0 | 0 | 0 | 0 | 1 | 0 |
| 3 | 1 | 0 | 0 | 1 | 0 | 0 | 0 | 1 | 0 | 0 | 0 | 0 | 0 | 0 | 0 | 0 |
| 4 | 8 | 0 | 0 | 0 | 0 | 0 | 0 | 0 | 0 | 0 | 0 | 0 | 0 | 0 | 0 | 0 |
| 5 | 5 | 0 | 0 | 0 | 0 | 0 | 1 | 0 | 0 | 0 | 0 | 0 | 1 | 3 | 0 | 0 |
| 6 | 0 | 0 | 0 | 1 | 1 | 0 | 2 | 6 | 0 | 0 | 0 | 0 | 0 | 0 | 0 | 0 |
| 7 | 1 | 0 | 0 | 0 | 0 | 1 | 0 | 0 | 0 | 0 | 0 | 0 | 0 | 3 | 1 | 0 |
| 8 | 0 | 0 | 0 | 0 | 0 | 0 | 0 | 0 | 0 | 0 | 0 | 0 | 0 | 0 | 0 | 0 |
| 9 | 2 | 2 | 0 | 0 | 0 | 0 | 0 | 0 | 0 | 0 | 0 | 0 | 0 | 3 | 0 | 0 |
| 10 | 2 | 0 | 0 | 0 | 0 | 1 | 1 | 0 | 0 | 0 | 1 | 0 | 0 | 0 | 2 | 0 |
| 11 | 4 | 0 | 0 | 0 | 0 | 0 | 0 | 0 | 0 | 1 | 0 | 0 | 2 | 7 | 0 | 0 |
| 12 | 1 | 1 | 0 | 0 | 0 | 0 | 1 | 0 | 1 | 0 | 0 | 0 | 0 | 0 | 0 | 1 |
| 13 | 1 | 0 | 1 | 0 | 0 | 0 | 0 | 4 | 0 | 0 | 0 | 0 | 0 | 0 | 0 | 0 |
| 14 | 2 | 0 | 3 | 0 | 0 | 1 | 0 | 0 | 0 | 0 | 0 | 0 | 3 | 4 | 0 | 0 |
| 15 | 1 | 0 | 0 | 0 | 0 | 0 | 0 | 3 | 0 | 0 | 0 | 0 | 0 | 1 | 0 | 0 |
| 16 | 1 | 0 | 0 | 0 | 0 | 0 | 0 | 0 | 0 | 0 | 0 | 1 | 20 | 0 | 0 | 0 |
| 17 | 7 | 2 | 0 | 0 | 0 | 0 | 0 | 1 | 0 | 0 | 0 | 0 | 0 | 0 | 0 | 0 |
| 18 | 1 | 0 | 0 | 0 | 0 | 1 | 1 | 0 | 1 | 0 | 0 | 0 | 0 | 0 | 3 | 0 |
| 19 | 3 | 0 | 0 | 0 | 0 | 0 | 1 | 0 | 0 | 0 | 0 | 0 | 0 | 0 | 0 | 0 |
| 20 | 0 | 0 | 0 | 0 | 0 | 0 | 0 | 0 | 0 | 0 | 0 | 0 | 0 | 0 | 0 | 0 |
| 21 | 2 | 0 | 0 | 0 | 0 | 18 | 7 | 11 | 3 | 0 | 0 | 0 | 0 | 0 | 1 | 0 |
| 22 | 0 | 1 | 0 | 0 | 0 | 0 | 0 | 3 | 0 | 0 | 0 | 0 | 1 | 0 | 1 | 0 |
| 23 | 1 | 0 | 0 | 0 | 0 | 3 | 0 | 0 | 1 | 0 | 0 | 0 | 0 | 0 | 0 | 0 |
| 24 | 3 | 0 | 0 | 1 | 0 | 0 | 1 | 2 | 0 | 1 | 0 | 0 | 0 | 0 | 0 | 0 |
| 25 | 6 | 0 | 1 | 0 | 0 | 0 | 0 | 0 | 0 | 0 | 0 | 0 | 0 | 0 | 0 | 0 |
| 26 | 0 | 0 | 0 | 1 | 0 | 4 | 0 | 1 | 0 | 0 | 0 | 0 | 0 | 0 | 0 | 0 |
| 27 | 2 | 0 | 1 | 0 | 1 | 1 | 0 | 2 | 0 | 0 | 0 | 0 | 1 | 12 | 0 | 0 |
| 28 | 0 | 0 | 0 | 0 | 0 | 0 | 0 | 0 | 0 | 0 | 0 | 0 | 0 | 0 | 1 | 0 |
| 29 | 1 | 0 | 0 | 0 | 0 | 2 | 1 | 1 | 0 | 0 | 0 | 0 | 0 | 3 | 0 | 0 |
| 30 | 0 | 0 | 0 | 0 | 0 | 0 | 0 | 5 | 0 | 0 | 0 | 0 | 0 | 0 | 0 | 0 |

* Species names correspond to: A – *Psophia crepitans*, B – *Crax alector*, C – *Crypturellus erythropus*, D – *Tinamus major*, E – *Didelphis marsupialis*, F – *Mazama americana*, G – *Mazama nemorivaga*, H – *Pecari tajacu*, I – *Tapirus terrestris*, J – *Eira barbara*, K – *Nasua nasua*, L – *Procyon cancrivorus*, M – *Cuniculus paca*, N – *Myoprocta acouchy*, O – *Dasyprocta* *leporina*, P – *Sciurus aestuans*.
